# Effectiveness of Mindfulness and Qigong Training for Self-Healing in patients with Hwabyung and depressive disorder: a randomized controlled trial

**DOI:** 10.3389/fpsyt.2025.1508937

**Published:** 2025-06-13

**Authors:** Seok-In Yoon, Hui-Yeong Park, Chan Park, Jiho Pyun, Jae-Hong Yoo, Geum-Ju Song, Hyun Woo Lee, Sun-Yong Chung, Jong Woo Kim

**Affiliations:** ^1^ Department of Neuropsychiatry, College of Korean Medicine, Kyung Hee University, Seoul, Republic of Korea; ^2^ Department of Neuropsychiatry, Kyung Hee University Korean Medicine Hospital at Gangdong, Seoul, Republic of Korea; ^3^ Department of Neuropsychiatry, Graduate School, Kyung Hee University, Seoul, Republic of Korea; ^4^ Industry-Academic Cooperation Foundation, Kyung Hee University, Seoul, Republic of Korea

**Keywords:** Hwabyung, depressive disorder, vitality, mindfulness, qigong, randomized controlled trial

## Abstract

**Background:**

Hwabyung is a Korean culture-bound syndrome characterized by anger-related physical and psychological symptoms. Depressive disorder is a common mental disorder that occurs worldwide and is highly comorbid with Hwabyung. In traditional East Asian medicine, both Hwabyung and depression are associated with an imbalance in vital energy termed qi. Mindfulness induces psychosomatic balance, and qigong facilitates the cultivation and regulation of qi, which may be effective treatments for Hwabyung and depression. This study aimed to investigate whether Mindfulness and Qigong Training for Self-Healing (MQT-SH) could improve psychosomatic symptoms in patients with Hwabyung and depressive disorders.

**Methods:**

This was a two-arm, randomized controlled trial. Patients diagnosed with both Hwabyung and depressive disorder were included in the trial. A total of 64 participants were recruited and randomly assigned to either the experimental (*n*=32) or control group (*n*=32). The experimental group underwent MQT-SH that consisted of mindfulness and qigong for the first six weeks, whereas the control group received no treatment. During the next six weeks, the control group performed MQT-SH for ethical equity. Assessments were conducted at baseline, post-intervention (6-week), and follow-up (12-week).

**Results:**

MQT-SH significantly decreased Hwabyung, depression, anxiety, and anger while increasing subjective vitality, and the effectiveness of the intervention were maintained at a short-term follow-up of 6 weeks. Physical vitality mediated the effectiveness of the MQT-SH on Hwabyung, whereas psychological vitality mediated the effectiveness of the MQT-SH on depression. Only one adverse event was reported during the intervention period. Home practice was not significantly associated with any intervention change.

**Discussion:**

This study demonstrated that MQT-SH is an effective and safe intervention for patients with Hwabyung and depressive disorders. This study explored the possibility that subjective vitality may serve as a mechanism for treating psychosomatic and mood symptoms. Future studies should control for placebo effects and conduct long-term follow-ups.

**Ethics and dissemination:**

This study was approved by the Institutional Review Board of Kyung-Hee University Oriental Medicine Hospital in Gangdong (KHNMCOH 2023-09-003).

**Clinical trial registration:**

https://cris.nih.go.kr/cris/search/detailSearch.do?seq=26882&status=5&seq_group=25511&search_page=M, identifier KCT0008937.

## 1 Introduction

Hwabyung is a culture-specific disorder characterized by unresolved and explosive symptoms of negative emotions such as anger (1). Hwabyung includes physical symptoms such as chest tightness and heat and psychological symptoms such as resentment and anger (2). Hwabyung is caused by specific stressful events (3). The diagnostic criteria for Hwabyung were established by Kim et al. (2). According to the standardized diagnostic criteria, the prevalence of Hwabyung in Koreans was reported to be 4.2–13.3% (4–7).

Depressive disorder is a mental illness characterized by the absence of positive emotions or a depressed mood (8). It negatively affects not only personal well-being but also social functioning including job performance (9). Depressive disorder is a common disorder worldwide, with nearly 330 million people diagnosed by 2021 (10). In the 2021 Global Burden of Disease Study, depressive disorders ranked 12th in terms of disease burden, similar to malaria, congenital defects, tuberculosis, and headache (11).

Although Hwabyung and depressive disorders have different diagnostic criteria, they often co-occur with each other. According to an epidemiological study, 44% of patients diagnosed with Hwabyung were diagnosed with depressive disorders (12). Considering that 28.5% of patients with depressive disorders have generalized anxiety disorder as a comorbidity (13), depressive disorder and Hwabyung may have very high comorbidities. Therefore, patients diagnosed with depressive disorders should be considered differential diagnosis and concomitant treatment for Hwabyung.

According to Donguibogam, a classic traditional East Asian medicine (TEAM), physical and psychological suffering occurs due to imbalance and disharmony of qi (14). For example, Hwabyung is a psychological blockage accompanied by physical symptoms, in which qi, called Hwa (火), is tilted upward and does not circulate properly (15). Depression is a state of psychological exhaustion that occurs when qi is deficient, its movement is weakened, and qi becomes stagnant (16). In this way, both Hwabyung and depression result from an imbalance (i.e., deficiency or stagnation) of qi. To address these problems, TEAM aims to achieve an optimal balance between mind and body by cultivating and regulating qi (17, 18).

Qi refers to the inherent life energy of an organism and is considered the root of vitality, energy, and spirit (18). Vitality is a broad and multidimensional concept that includes energy, physical and psychological health, quality of life, and wellbeing (19, 20). Vitality is an important predictor of both physical and psychological health. Previous studies have demonstrated that subjective vitality serves as an important predictor of health and well-being, much like objective indicators such as disease history, medication use, functional impairment (21), and it may reduce the risk of coronary heart disease (22), new disability, and mortality (23). Other studies have demonstrated that subjective vitality functions as a buffer against stressful events (24, 25)

Recently, non-pharmacological interventions such as mindfulness and qigong have been utilized to treat mental illness while minimizing the side effects of medication (26–28). Mindfulness is defined as an intentional state of non-judgmental attention focused on the present moment (29, 30). Mindfulness induces a relaxation response (31), helps to regulate emotions such as depression and anger (32, 33), and promotes cognitive reappraisal (34). Standardized mindfulness-based programs have been developed to regulate negative affect in patients with chronic diseases and depressive disorders (32, 35). Previous studies have demonstrated that mindfulness programs are effective in alleviating the symptoms of Hwabyung, depression, anxiety, and anger (33, 36–38).

Qigong is a Taoist-based practice that cultivates and regulates vital energy, termed qi, through body postures and movements, deep breathing, and mental concentration (39). Qigong is a broad concept that includes both static and dynamic practices and encompasses various traditional practices, such as Ba Duan Jin, Liu Zi Jue, Wu Qin Xi, and Kouksundo (40). In TEAM, cultivating and regulating qi is considered a therapeutic mechanism for alleviating mental and physical symptoms (14, 17). Previous studies have demonstrated that qigong significantly improves vitality (41) and reduces Hwabyung, depression, anxiety, and anger (42–44).

Qigong is closely associated with mindfulness. Qigong is practiced with meditative attention called mindfulness. Qigong emphasizes calm and focused attention to be aware of qi as a phenomenological experience and to regulate and manipulate it. The specific attention of qigong is similar to the three characteristics of mindfulness, including present attention, nonjudgmental attitude, and intention (29, 30). Furthermore, qi can be fully cultivated when accompanied by mindfulness (18, 45). In this context, qigong is called a mindful movement or exercise (46, 47).

Qigong shares some therapeutic mechanisms with mindfulness (48). Mindfulness induces homeostasis of the autonomic nervous system through a physiological relaxation response (31). Mindfulness helps alleviate cognitive biases (49, 50) and regulate emotional problems (33) through nonjudgmental focused attention. These functions of mindfulness are consistent with the therapeutic mechanisms (i.e., balance and harmony) of qigong emphasized in TEAM (18). However, qigong has a more active goal than does mindfulness, in that it cultivates and regulates vital energy.

The Mindfulness and Qigong Training for Self-Healing (MQT-SH) is a standardized mind-body intervention program based on traditional Korean medicine (17). The MQT-SH integrates mindfulness and qigong to enhance an organism’s self-healing ability. The first module consisted of mindfulness-based trainings for maintaining physical and psychological homeostasis. The second module consisted of qigongs to mindfully recognize, cultivate, and regulate qi. Qigong is a broad concept that encompasses many different traditional practices (40), making it difficult to standardize qigong-related programs. To address these limitations and increase the reproducibility of future studies, the authors operationally defined qigong as feeling, accumulating, and utilizing sensory experiences called qi (17).

This trial aimed to investigate the effectiveness of the MQT-SH in patients with Hwabyung and depressive disorders. The primary hypothesis was that the experimental group (EG) would have significantly reduced Hwabyung and depression compared to that of the control group (CG). This may provide empirical evidence regarding whether MQT-SH can help to alleviate the main psychosomatic symptoms of patients with Hwabyung and depressive disorders. The secondary hypothesis was that the EG would significantly reduce anxiety and anger while significantly improving subjective vitality compared to the CG. This may demonstrate whether MQT-SH is effective in addressing concomitant emotional problems in patients with Hwabyung and depressive disorder and further demonstrate the effectiveness of MQT-SH on their quality of life. In addition, a mediation analysis was performed to explore the therapeutic mechanisms (e.g., subjective vitality) of the MQT-SH, which will provide validity of the TEAM approach in the treatment of Hwabyung and depressive disorder.

Additionally, this study aimed to investigate the effects of home practices on intervention changes. Home practice is considered an important element in inducing the intended therapeutic change in modern psychotherapy, which requires active patient cooperation (51). Although a growing number of studies have explored the relationship between home practice and intervention change, it is still in its early stages, and conflicting results have been reported. A meta-analysis found that the correlation between home practice and intervention change was significant but small (*r* = .26), and 21 of 28 studies did not identify a significant relationship between home practice and intervention change (51). Carmody and Baer (52) demonstrated that home practice time is significantly associated with mindfulness, perceived stress, and psychological symptoms, whereas Davidson et al. (53) and Kim and Jeon (54) found that the frequency and duration of home practice are not significantly associated with physical or psychological symptoms. Further replication studies are needed to determine the effects of home practice. Therefore, this study investigated the effects of home practice on the MQT-SH outcomes.

## 2 Methods

### 2.1 Design

**Figure 1:**
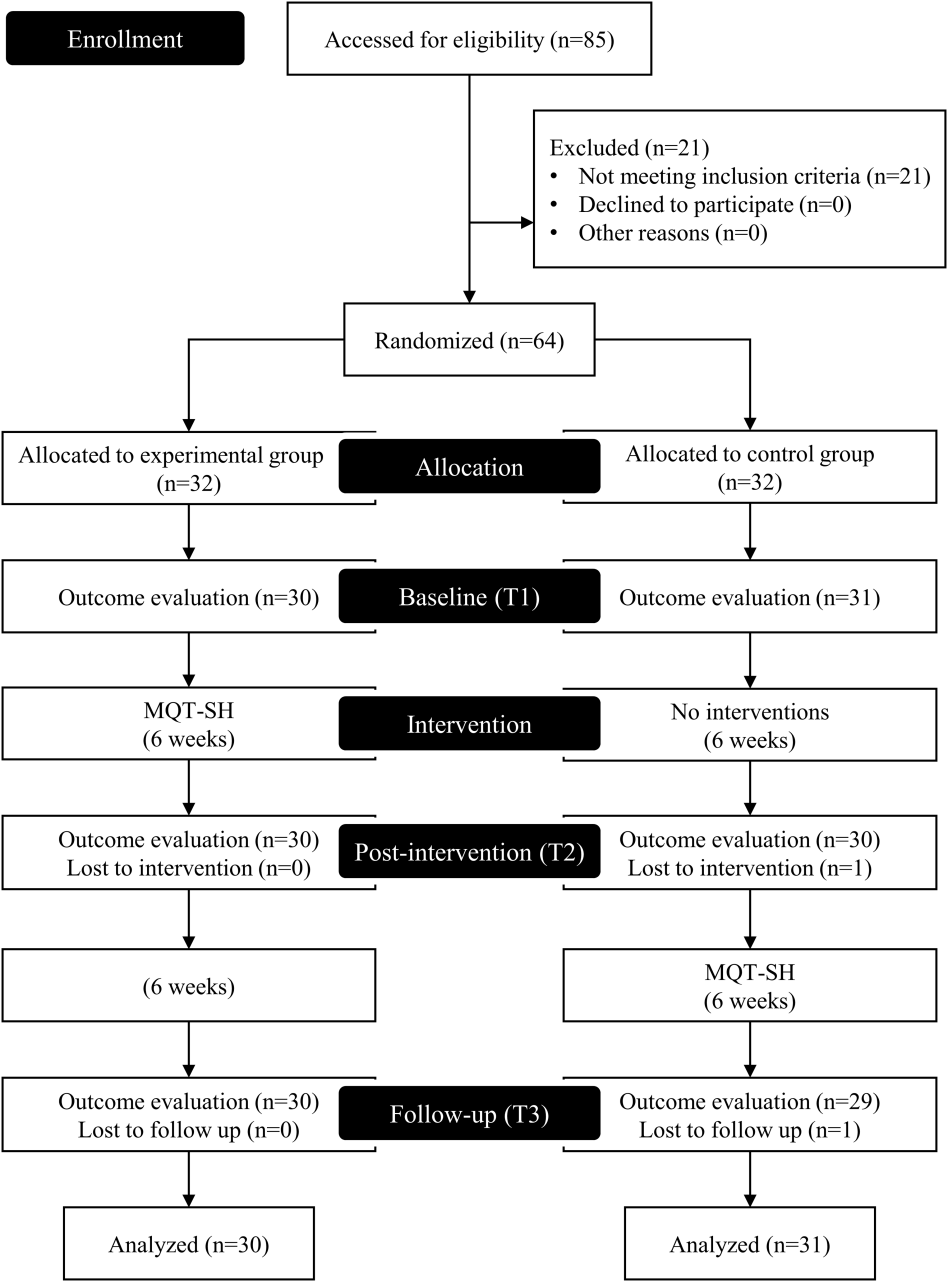
Study flow chart.

This trial was designed as a two-arm, block randomized controlled trial. Patients were assigned to either the EG or the CG. The trial was conducted over 12 weeks. During the first six weeks, the EG received MQT-SH, whereas the CG received no treatment as a wait-list control. During the next six weeks, the CG received MQT-SH with consideration for ethical equity. Assessments were performed at three points: baseline (T1), post-intervention (T2, 6 weeks), and follow-up (T3, 12 weeks). **Figure 1** shows an overview of the study design.

This trial was conducted at a single center, the Kyung Hee University Korean Medicine Hospital in Gangdong, Republic of Korea. Participants were enrolled between November 31, 2023, and May 31, 2024. The first participant completed the trial on January 31, 2024, and the last participant completed the trial on August 31, 2024.

This trial was approved by the Institutional Review Board (IRB) of Kyung-Hee University Korean Medicine Hospital in Gangdong (KHNMCOH 2023-09-003). This trial was registered with the Clinical Research Information Service (CRIS) of the Republic of Korea (KCT0008937). This trial followed a published study protocol (55) and any changes to the study plan were reviewed in advance by the IRB.

### 2.2 Participants

The participants were recruited through advertisements in hospitals and communities. Potential participants interested in participating were informed about the study. Written informed consent was obtained from all participants before their inclusion in the study. Participants were included if they i) had simultaneously been diagnosed with Hwabyung and depressive disorder by the Hwa-Byung Diagnostic Interview Schedule (HBDIS; 2) and Structured Clinical Interview for DSM-5 (SCID-5; 56); ii) are ≥ 19 years of age; and iii) can participate in the study by considering the timetable. Participants were excluded if they i) had hallucinations or delusions; ii) had organic brain disorders such as major cognitive impairment (e.g., dementia), epilepsy, intellectual disability, or personality disorder; iii) had a condition that made it difficult to complete the interview and questionnaire tests conducted in this study (e.g., difficulty reading, writing, listening, speaking, or understanding); and iv) had changes in medications such as antidepressants within the past month.

The sample size was estimated using G* Power (57). The sample size calculation was based on a power of 80%, type I error of 5%, and a small to medium effect size (η^2^ = 0.04) for the intervention. The effect size (Cohen’s *d* = 0.48) was referenced from a meta-analysis of qigong training for depression (58). We estimated a minimum sample size of 25 participants per group (50 participants). Considering a dropout rate of 20%, 64 participants were enrolled, with 32 allocated to each group.

### 2.3 Intervention

The EG underwent MQT-SH during the first six weeks. **Table 1** lists the detailed procedures for MQT-SH. The MQT-SH is a standardized mindfulness- and qigong-based program aimed at achieving optimal physical and psychological conditions (17). The MQT-SH manual was developed to implement standardized interventions. In TEAM, excessive emotions lead to physical and psychological distress (59), and problems of imbalance and disharmony can be overcome through the circulation and interchange of qi (60). Accordingly, MQT-SH consists of two modules: i) balance and harmony and ii) circulation and interchange.

**Table 1:** Intervention sessions.

Module	Session	Subject	Education (20 min)	Practice (40 min)	Group activities (30 min)
Balance and harmony	1	Breathing	Understanding breathing How to use breathing	Breathing meditation	Checking home practice; Sharing experiences; Question and answer
	2	Relaxation	Balance and harmony of stress and relaxation	Autogenic training; Body scan	
	3	Mindfulness	Understanding mindfulness	Sitting meditation (breathing and bodily sensations)	
Circulation and interchange	4	Feeling Qi	Energy that can be experienced phenomenologically	Meditation with Qi 1) Breathing meditation 2) Body scan 3) Mindfulness of Qi	Checking home practice; Sharing experiences; Question and answer
	5	Accumulating Qi	Two ways to obtain energy (e.g. breathing and eating)	Danjeon breathing; Eating meditation	
	6	Utilizing Qi	How to use energy	Qigong movements; Walking meditation	

The first module, Balance and Harmony, comprised the 1st to 3rd sessions. This module emphasizes the balance of breathing (e.g., inhalation and exhalation), autonomic nervous system (e.g., sympathetic and parasympathetic nervous systems), and mind (e.g., past, present, and future) for health. This module taught mindfulness training to promote healthy balance and harmony. In the 1st session, participants were taught the meaning of breathing and how to control their breathing. They then practiced breathing meditation. Breathing meditation involves counting numbers or adding phrases while focusing on breathing. In the 2nd session, the participants were educated on the balance between relaxation and stress. After the education, they practiced autogenic training and body scans. In the 3rd session, the participants were taught mindfulness, followed by sitting meditation. Sitting meditation emphasizes the awareness of inner experiences, such as breathing and bodily sensations.

The second module, Circulation and Interchange, consists of the 4th to 6th sessions. This module teaches qigong to promote the optimal circulation and interchange of qi. In MQT-SH, qigong is operationally defined in three ways: feeling, accumulating, and utilizing sensory energy (qi). In the 4th session, participants were taught qi, which can be experienced phenomenologically, and then practiced meditation with qi. Meditation with qi aims to sensuously experience qi in a relaxed and calm state. Meditation with qi consists of breathing meditation, body scans, and mindfulness of qi. In the 5th session, the participants learned two ways to obtain vital energy: breathing and eating. They practiced danjeon breathing and eating meditations. In the 6th session, participants were taught how to use vital energy and practiced brief qigong movements and walking meditation.

The intervention was delivered as a group program, with six or eight participants per group. The standard MQT-SH is a program consisting of eight sessions, 3 hours per session (17), but this trial was designed as a short-form program considering time and space constraints. In this trial, the MQT-SH was conducted once a week for 1.5 h for a total of six weeks. Each session consisted of 20 minutes of education, 40 minutes of guided practice (e.g., mindfulness and qigong), and 30 minutes of group activities (e.g., checking home practice and sharing practice experiences). The guided practice was conducted using audio recordings. The participants were asked to practice at home at least five times a week for approximately 30 minutes per day. Participants were provided with guided audio recordings for home practice.

In contrast, the CG received no treatment during the first six weeks. Considering ethical equity, the CG received the same MQT-SH for the next six weeks.

### 2.4 Randomization

Participants were randomly allocated to either the EG or the CG (1:1 ratio, block size = 4). The random number table for allocation was computer generated and managed by a researcher (YSI). Before enrollment, the participants and other researchers (e.g., assessors and instructors) were unaware of the allocation. After enrollment, the YSI allocated participants to the EG or CG according to random numbers, and the allocation results were shared only with the clinical research coordinator.

### 2.5 Blinding

In this clinical trial, the assessors were blinded to the intervention group and had no contact or conversation with the participants except for the clinical interview. However, participants and instructors were not blinded due to the nature of the interventions.

### 2.6 Measurements

#### 2.6.1 Primary outcomes

Primary outcomes were assessed at T1, T2, and T3.

The Hwabyung Scale (HS; 61) and Hwabyung Comprehensive Test (HCT; 62) were used to assess Hwabyung-related personality and symptoms. The HS is a self-report 5-point (0-4) Likert scale consisting of 16 items on Hwabyung personality and 15 items on Hwabyung symptoms. Higher HS scores indicated greater Hwabyung-related personality traits and symptoms. In a previous study (61), Cronbach’s alpha for the HS was 0.85 for Hwabyung personality, 0.93 for Hwabyung symptoms, and 0.93 overall.

The HCT is a self-report 5-point (0-4) Likert scale consisting of 13 items on Hwabyung symptoms and 21 items on Hwabyung personality. A higher HCT score indicated greater Hwabyung-related personality traits and symptoms. In a previous study (62), Cronbach’s alphas for the HCT was 0.89 for Hwabyung symptoms and 0.95 for Hwabyung personality.

The Korean version of the Hamilton Depression Rating Scale (HDRS; 63) was used to assess depressive symptoms. The HDRS is a 17-item observer rating scale. Each item is scored from 0 to 2 or from 0 to 4. The total score ranged from 0 to 52. Higher HDRS scores indicated more severe depressive symptoms. A structured interview manual was used to increase inter-rater reliability (64). The raters were blinded to the allocations.

#### 2.6.2 Secondary outcomes

Secondary outcomes were assessed at T1, T2, and T3.

The Hamilton Anxiety Rating Scale (HARS; 65) was used to assess anxiety symptoms. The HARS is a 14-item observer-rating scale. Each item is scored from 0 to 4. The total score ranged from 0 to 56. Higher HARS scores indicated more severe anxiety symptoms. A structured interview manual was used to increase inter-rater reliability (66). The raters were blinded to the allocations.

The Korean Adaptation of the State-Trait Anger Expression Inventory (STAXI; 67) was used to assess anger experiences and expressions. The STAXI is a self-reported 4-point (1-4) Likert scale consisting of 10 items on state anger, 10 items on trait anger, and 24 items on anger expression. Anger expressions consisted of anger-out, anger-in, and anger controls. A higher STAXI score indicated greater anger-related experiences and expressions. In a previous study (67), Cronbach’s alpha was 0.90 for state anger, 0.75 for trait anger, 0.70 for anger-out, 0.66 for anger-in and, 0.79 for anger-control.

The Integrative Vitality Scale (IVS; 68) was used to assess subjective vitality. The IVS operationally defines qi as two subfactors, including physical and psychological vitality. Physical vitality is a positive physical experience through sufficient relaxation and rest, whereas psychological vitality is a state of interest and enjoyment in life and active engagement in it. According to Yoon et al. (68), integrative vitality refers to a state in which both physical and psychological vitality increase and relaxation (rest) and awakening (engagement) are balanced. The IVS is a self-reported 5-point (0-4) Likert scale consisting of 11 items on physical vitality and 11 on psychological vitality. A higher IVS score indicated a higher level of subjective vitality. In a previous study (68), Cronbach’s alpha was 0.91 for physical vitality, 0.91 for psychological vitality, and 0.94 overall.

#### 2.6.3 Adherence

Adherence refers to the extent to which patients actively follow medical advice. Adherence was assessed on the basis of attendance, home practices, and dropouts. Attendance refers to the total attendance frequency per participant and ranges from 0 to 6. Before each MQT-SH session, the instructor checked the participants’ attendance for each session.

The participants were asked to practice at home after each session. The participants were asked to record the date, start time, and end time of each home practice session. Records were reviewed weekly. The total frequency and duration of home practice were both calculated based on the participants’ records over a 6-week period. The total frequency of home practice refers to the total number of home practice sessions recorded by a participant. The total duration of the home practice was calculated as the sum of the differences between the end and start times of each home practice session.

Dropouts were defined as i) individuals who attended three or fewer of the six sessions; ii) individuals who complained of physical or psychological discomfort and wanted to give up because of such discomfort; or iii) individuals who wanted to give up for reasons other than discomfort.

#### 2.6.4 Adverse events

Adverse events were assessed by type, frequency, and severity. Severity was assessed using three grades (mild, moderate, or severe). Mild was defined as an adverse event that did not interfere with the activities of daily living. Moderate is defined as an adverse event that interferes with daily life, but is not dangerous. Severe is defined as an adverse event that is serious and interferes with basic daily activities (e.g., eating and changing clothes). Adverse events were collected weekly by a clinical research coordinator. When adverse events were reported by participants, it was monitored daily until the event was resolved.

### 2.7 Statistical analyses

SPSS version 22.0, and PROCESS Macro for SPSS were used. Analyses based on the intention-to-treat principle were conducted with missing data imputed using the expectation-maximization algorithm.

To verify baseline homogeneity across the groups, *t*-tests and chi-square tests were performed. An analysis of variance (ANOVA) was performed to investigate the effectiveness of MQT-SH. When the baseline homogeneity of the outcome variables was verified, a 2 (group: experimental vs. control) × 2 (time: T1 vs. T2) repeated-measures ANOVA was performed. If the interaction effect between the group and time was significant, a simple main-effect analysis was performed. In contrast, when baseline homogeneity was not verified, one-way analysis of covariance (ANCOVA) was performed with the T1 outcome variables as covariates.

Additional analyses were also performed. First, to investigate the follow-up effects of the MQT-SH, a paired *t*-test was performed to compare T3 and T1, and T3 and T2 in the EG. Second, a paired *t*-test comparing T3 and T2 in the CG was performed to investigate the effectiveness of the MQT-SH. Third, mediation analysis was performed to explore the therapeutic mechanism of MQT-SH. The independent variable is group (CG = 0 vs. EG = 1), the dependent variable is the T2 − T1 change in Hwabyung or depressive symptoms, and the mediator is the T2 − T1 change in physical and psychological vitality. Fourth, correlation analysis was performed to investigate the effects of home practices on intervention changes. The pre- and post-intervention changes in all outcomes were calculated (Post – Pre). Data from both the EG and CG were used for the correlation analysis.

## 3 Results

### 3.1 Adherence to intervention

**Table 2** presents the participants’ adherence to the MQT-SH. In the EG and CG, two and three participants, respectively, dropped out of the study. Of these, two participants in the EG and one in the CG dropped out before T1 and were excluded from the analysis. In all sessions, the attendance rates were over than 80% in both the EG and CG. Attendance in the EG was significantly higher than that in the CG. There were no significant differences between the EG and CG in terms of the total frequency and duration of home practices.

**Table 2:** Attendance, home practice, and dropout of MQT-SH.

Variable	Mean ± SD or Rate% (*n*)		*t*/*χ* ^2^	*p*
	Experimental group (n = 30)	Control group (n = 31)		
Attendance	5.87 ± 0.35	5.38 ± 1.15	−2.23	0.032
Home practice				
Frequency	30.90 ± 10.39	31.81 ± 17.06	0.25	0.804
Duration	672.90 ± 335.58	760.58 ± 334.77	1.01	0.318
Clinical trial status^a^				
Completion	93.75 (30)	90.63 (29)	0.22	0.641
Dropout	6.25 (2)	9.38 (3)		

*^a^*, Clinical trial status was calculated based on the experimental group (n=32) and the control group (n=32).

### 3.2 Baseline characteristics

**Table 3** shows the baseline characteristics of the two groups. No significant differences were found between the two groups in terms of gender, age, or anger. However, there were significant differences between the two groups in terms of Hwabyung, depression, anxiety, and subjective vitality.

**Table 3:** Baseline characteristics of the participants.

Variable	Mean ± SD or Rate% (*n*)		*t*/*χ* ^2^	*p*
	Experimental group (*n* = 30)	Control group (*n* = 31)		
Gender			0.81	0.367
Male	13.33 (4)	6.45 (2)		
Female	86.67 (26)	93.55 (29)		
Age	53.37 ± 5.58	56.29 ± 7.72	−1.69	0.096
HS-personality	42.67 ± 6.24	39.19 ± 8.25	1.85	0.069
HS-symptoms	40.93 ± 8.01	34.48 ± 8.61	3.03	0.004
HCT-personality	57.37 ± 12.69	45.68 ± 12.50	3.63	<0.001
HCT-symptoms	36.60 ± 6.89	29.97 ± 7.51	3.59	<0.001
HDRS	24.83 ± 3.97	21.32 ± 4.29	3.31	0.002
HARS	27.60 ± 8.52	23.23 ± 7.12	2.18	0.033
STAXI-state	22.07 ± 8.27	19.39 ± 5.38	1.50	0.141
STAXI-trait	25.87 ± 5.71	23.97 ± 5.83	1.29	0.204
STAXI-out	16.27 ± 3.69	16.29 ± 4.28	−0.02	0.982
STAXI-in	21.83 ± 4.91	19.45 ± 5.35	1.81	0.075
STAXI-control	20.30 ± 4.34	20.16 ± 4.42	0.12	0.902
IVS-total	19.33 ± 10.42	27.77 ± 11.36	−3.02	0.004
IVS-phys.	8.43 ± 4.31	11.94 ± 6.19	−2.57	0.013
IVS-psychol.	10.90 ± 8.54	15.83 ± 6.55	−2.54	0.014

HS, Hwabyung Scale; HCT, Hwabyung Comprehensive Test; HDRS, Hamilton Depression Rating Scale; HARS, Hamilton Anxiety Rating Scale; STAXI; State-Trait Anger Expression Inventory; IVS, Integrative Vitality Scale.

### 3.3 Effectiveness of intervention

**Table 4** shows the means and standard deviations of the outcome variables by group and time, and provides the results of the ANOVA. For the HS-personality and STAXI, for which baseline homogeneity across the groups was verified, a 2 (group: experimental vs. control) × 2 (time: T1 vs. T2) repeated-measures ANOVA was conducted. In contrast, for the HS-symptoms, HCT, HDRS, HARS, and IVS, for which baseline homogeneity was violated, a one-way ANCOVA was conducted.

**Table 4:** Results of ANOVA (Mean ± SD).

Variable	Group	Baseline (T1)	Post-intervention (T2)	T1 to T2		
				ANOVA		*d*
				F	p	
HS-personality	EG (*n* = 30)	42.67 ± 6.24	36.13 ± 9.55	3.21^a^	0.078	0.91
	CG (*n* = 31)	39.19 ± 8.25	36.15 ± 10.21			0.38
HS-symptoms	EG (*n* = 30)	40.93 ± 8.01	28.97 ± 12.74	10.60^b^	0.002	1.09
	CG (*n* = 31)	34.48 ± 8.61	31.75 ± 9.95			0.40
HCT-personality	EG (*n* = 30)	57.37 ± 12.69	41.43 ± 15.47	6.84^b^	0.011	1.16
	CG (*n* = 31)	45.68 ± 12.50	40.76 ± 14.47			0.49
HCT-symptoms	EG (*n* = 30)	36.60 ± 6.89	25.20 ± 11.27	13.06^b^	<0.001	1.11
	CG (*n* = 31)	29.97 ± 7.51	29.32 ± 9.00			0.09
HDRS	EG (*n* = 30)	24.83 ± 3.97	12.13 ± 4.62	56.49^b^	<0.001	2.25
	CG (*n* = 31)	21.32 ± 4.29	20.93 ± 5.22			0.07
HARS	EG (*n* = 30)	27.60 ± 8.52	12.03 ± 7.10	48.88^b^	<0.001	1.51
	CG (*n* = 31)	23.23 ± 7.12	22.05 ± 5.69			0.22
STAXI-state	EG (*n* = 30)	22.07 ± 8.27	15.27 ± 7.22	10.54^a^	0.002	0.99
	CG (*n* = 31)	19.39 ± 5.38	17.70 ± 5.01			0.32
STAXI-trait	EG (*n* = 30)	25.87 ± 5.71	21.00 ± 5.42	15.02^a^	<0.001	1.08
	CG (*n* = 31)	23.97 ± 5.83	23.15 ± 5.08			0.23
STAXI-out	EG (*n* = 30)	16.27 ± 3.69	14.07 ± 3.56	1.81^a^	0.183	0.56
	CG (*n* = 31)	16.29 ± 4.28	15.24 ± 4.12			0.34
STAXI-in	EG (*n* = 30)	21.83 ± 4.91	17.97 ± 4.70	11.07^a^	0.002	0.88
	CG (*n* = 31)	19.45 ± 5.35	19.01 ± 5.81			0.12
STAXI-control	EG (*n* = 30)	20.30 ± 4.34	18.86 ± 4.07	3.65^a^	0.061	0.49
	CG (*n* = 31)	20.16 ± 4.42	20.08 ± 3.39			0.03
IVS-total	EG (*n* = 30)	19.33 ± 10.42	37.00 ± 19.07	12.57^b^	<0.001	1.00
	CG (*n* = 31)	27.77 ± 11.36	30.78 ± 12.17			0.43
IVS-phys.	EG (*n* = 30)	8.43 ± 4.31	18.27 ± 10.44	9.37^b^	0.003	0.91
	CG (*n* = 31)	11.94 ± 6.19	13.53 ± 5.86			0.31
IVS-psychol.	EG (*n* = 30)	10.90 ± 8.54	18.73 ± 10.14	9.29^b^	0.003	0.89
	CG (*n* = 31)	15.83 ± 6.55	17.25 ± 7.05			0.42

^a^, Interaction effects in Group × Time repeated measures ANOVA; ^b^, Group effects in one-way ANCOVA; EG, experimental group; CG, control group; HS, Hwabyung Scale; HCT, Hwabyung Comprehensive Test; HDRS, Hamilton Depression Rating Scale; HARS, Hamilton Anxiety Rating Scale; STAXI; State-Trait Anger Expression Inventory; IVS, Integrative Vitality Scale.

Additionally, paired *t*-tests were performed to verify the follow-up effects in the EG and the post-intervention effects in the CG. **Tables 5**, **6** present the results of the paired *t*-test for the EG and CG, respectively.

**Table 5:** Follow-up effects of MQT-SH in the experimental group (mean ± SD).

Variable	Pre (T1)	Post (T2)	Follow-up (T3)	T1 to T3			T2 to T3		
				*t*	*p*	*d*	*t*	*p*	*d*
HS-personality	42.67 ± 6.24	36.13 ± 9.55	35.80 ± 9.03	−4.66	<0.001	0.85	−0.27	0.791	0.05
HS-symptoms	40.93 ± 8.01	28.97 ± 12.74	28.00 ± 9.95	−8.00	<0.001	1.46	−0.45	0.656	0.08
HCT-personality	57.37 ± 12.69	41.43 ± 15.47	41.43 ± 16.09	−6.02	<0.001	1.10	0.00	1.000	0.00
HCT-symptoms	36.60 ± 6.89	25.20 ± 11.26	24.40 ± 9.63	−8.60	<0.001	1.57	−0.48	0.638	0.09
HDRS	24.83 ± 3.97	12.13 ± 4.62	14.47 ± 5.35	−11.16	<0.001	2.04	2.21	0.035	0.41
HARS	27.60 ± 8.52	12.03 ± 7.10	13.80 ± 6.47	−8.19	<0.001	1.50	1.32	0.198	0.24
STAXI-state	22.07 ± 8.27	15.27 ± 7.22	14.90 ± 6.15	−6.69	<0.001	1.22	−0.38	0.704	0.07
STAXI-trait	25.87 ± 5.71	21.00 ± 5.42	20.93 ± 4.46	−6.77	<0.001	1.24	−0.10	0.922	0.02
STAXI-out	16.27 ± 3.69	14.07 ± 3.56	13.60 ± 3.19	−3.93	<0.001	0.72	−0.70	0.489	0.13
STAXI-in	21.83 ± 4.91	17.97 ± 4.70	17.87 ± 4.83	−4.81	<0.001	0.88	−0.17	0.866	0.03
STAXI-control	20.30 ± 4.34	18.86 ± 4.07	18.47 ± 2.87	−2.99	0.006	0.55	−0.72	0.478	0.13
IVS-total	19.33 ± 10.42	37.00 ± 19.07	29.73 ± 14.65	4.67	<0.001	0.85	−2.28	0.030	0.42
IVS-phys.	8.43 ± 4.31	18.27 ± 10.04	13.77 ± 6.51	3.91	<0.001	0.71	−2.65	0.013	0.49
IVS-psychol.	10.90 ± 8.54	18.73 ± 10.14	15.97 ± 9.17	4.25	<0.001	0.78	−1.63	0.113	0.30

MQT-SH, Mindfulness and Qigong Training for Self-Healing; HS, Hwabyung Scale; HCT, Hwabyung Comprehensive Test; HDRS, Hamilton Depression Rating Scale; HARS, Hamilton Anxiety Rating Scale; STAXI, State-Trait Anger Expression Inventory; IVS, Integrative Vitality Scale

**Table 6:** Effectiveness of MQT-SH in the control group (mean ± SD).

Variable	Pre (T2)	Post (T3)	T2 to T3		
			*t*	*p*	*d*
HS-personality	36.15 ± 10.21	33.48 ± 10.53	−1.27	0.215	0.23
HS-symptoms	31.75 ± 9.95	23.77 ± 10.33	−3.31	0.002	0.60
HCT-personality	40.76 ± 14.47	32.68 ± 13.87	−2.97	0.006	0.53
HCT-symptoms	29.32 ± 5.22	19.12 ± 9.62	−4.89	<0.001	1.03
HDRS	20.93 ± 5.22	11.25 ± 6.48	−7.93	<0.001	1.43
HARS	22.05 ± 5.69	12.12 ± 8.24	−6.75	<0.001	1.21
STAXI-state	17.70 ± 5.01	12.69 ± 3.14	−4.50	<0.001	0.81
STAXI-trait	23.15 ± 5.08	19.52 ± 5.12	−4.08	<0.001	0.73
STAXI-out	15.24 ± 4.12	13.15 ± 3.96	−0.99	0.330	0.54
STAXI-in	19.01 ± 5.81	16.38 ± 5.70	−3.97	<0.001	0.71
STAXI-control	20.08 ± 3.39	19.36 ± 4.66	−3.04	0.005	0.18
IVS-total	30.78 ± 12.17	48.84 ± 19.98	5.56	<0.001	0.95
IVS-phys.	13.53 ± 5.86	23.58 ± 10.37	4.48	<0.001	1.00
IVS-psychol.	17.25 ± 7.05	25.27 ± 10.24	5.27	<0.001	0.81

MQT-SH, Mindfulness and Qigong Training for Self-Healing; HS, Hwabyung Scale; HCT, Hwabyung Comprehensive Test; HDRS, Hamilton Depression Rating Scale; HARS, Hamilton Anxiety Rating Scale; STAXI; State-Trait Anger Expression Inventory; IVS, Integrative Vitality Scale.

#### 3.3.1 Hwabyung

For HS-personality, repeated-measures ANOVA did not show a significant interaction effect between group and time. Because the interaction effect was not significant, a simple main-effect analysis was not performed.

The ANCOVA showed that HS-symptoms at T2 were significantly lower in the EG than in the CG. ANCOVA showed that the HCT-personality and symptoms at T2 were significantly lower in the EG than in the CG.

The paired *t*-test showed that in the EG, the HS and HCT at T3 were significantly lower than those at T1 but did not significantly differ from those at T2. In the CG, the HS-symptoms and HCT at T3 were significantly lower than those at T2.

#### 3.3.2 Depression

ANCOVA showed that the HDRS score at T2 was significantly lower in the EG than in the CG.

The paired *t*-test showed that in the EG, the HDRS score at T3 was significantly lower than that at T1 but significantly higher than that at T2. In the CG, the HDRS score at T3 was significantly lower than that at T2.

#### 3.3.3 Anxiety

ANCOVA showed that the HARS score at T2 was significantly lower in the EG than in the CG.

The paired *t*-test showed that in the EG, the HARS score at T3 was significantly lower than that at T1 but did not significantly differ from that at T2. In the CG, the HARS score at T3 was significantly lower than that at T2.

#### 3.3.4 Anger

For the STAXI-state, STAXI-trait, and STAXI-in, repeated-measures ANOVA revealed a significant interaction effect between group and time. Simple main-effect analysis showed that in the EG, the STAXI-state at T2 was significantly lower than that at T1, *F* = 36.68, *p* < 0.001, whereas in the CG, the STAXI-state did not show a significant difference between T1 and T2, *F* = 2.33, *p* = 0.132. In the EG, the STAXI-trait at T2 was significantly lower than that at T1, *F* = 42.64, *p* < 0.001, whereas in the CG, the STAXI-trait did not show a significant difference between T1 and T2, *F* = 1.24, *p* = 0.270. In the EG, STAXI-in at T2 was significantly lower than that at T1, *F* = 27.82, *p* < 0.001, whereas in the CG, STAXI-in did not show a significant difference between T1 and T2, *F* = 0.38, *p* = 0.540.

For the STAXI-out and control, the repeated-measures ANOVA did not show a significant interaction effect between group and time. Because the interaction effect was not significant, a simple main-effect analysis was not performed.

The paired *t*-test showed that in the EG, the STAXI at T3 was significantly lower than that at T1 but did not significantly differ from that at T2. In the CG, the STAXI at T3 was significantly lower than that at T2, except for STAXI-out.

#### 3.3.5 Subjective vitality

The ANCOVA showed that the IVS-total, phys., and psychol. at T2 were significantly higher in the EG than in the CG.

The paired *t*-test showed that in the EG, the IVS-total, and phys. at T3 were significantly higher than those at T1 but were significantly lower than those at T2. In the EG, the IVS-psychol. at T3 was significantly higher than that at T1 but did not significantly differ from that at T2. In the CG, the IVS at T3 was significantly higher than that at T2.

### 3.4 Therapeutic mechanism of intervention

The parallel mediation effects of physical and psychological vitality on the relationship between MQT-SH and Hwabyung or depression were investigated (**Supplementary Materials 1**, **2**). For HS-symptoms, the total and mediation effects of the IVS-phys. and IVS-psychol. were significant; however, the direct effect was not. For HCT-symptoms, the total, direct, and the mediation effects of IVS-phys. were significant; however, the mediation effect of IVS-psychol. was not significant. For the HDRS, the total, direct, and mediation effects of the IVS -psychol. were significant; however, the mediation effect of IVS-phys. was not significant.

### 3.5 Effects of home practice on the intervention change

**Table 7** presents Pearson correlation coefficients between intervention change and home practice. Neither the total frequency nor the duration of home practice were significantly correlated with intervention change. Total frequency of home practice tended to be correlated with HARS change, but the correlation coefficient was very low.

**Table 7:** Pearson correlation coefficients between home practice and intervention changes (*n* = 61).

Variable^a^	Frequency	Duration
HS-personality	−0.12	−0.16
HS-symptoms	−0.07	−0.20
HCT-personality	−0.02	−0.03
HCT-symptoms	−0.07	−0.07
HDRS	−0.02	−0.05
HARS	−0.24	−0.17
STAXI-state	0.09	−0.01
STAXI-trait	−0.08	−0.15
STAXI-out	−0.04	−0.12
STAXI-in	−0.02	−0.04
STAXI-control	0.11	0.05
IVS-total	−0.08	−0.06
IVS-phys.	−0.03	−0.07
IVS-psychol.	−0.12	−0.04

a , Outcomes were calculated by subtracting pre-values ​​(experimetal group=T1, control group=T2) from post-values ​​(experimetal group=T2, control group=T3); HS, Hwabyung Scale; HCT, Hwabyung Comprehensive Test; HDRS, Hamilton Depression Rating Scale; HARS, Hamilton Anxiety Rating Scale; STAXI; State-Trait Anger Expression Inventory; IVS, Integrative Vitality Scale.

All coefficients are not significant.

### 3.6 Safety of intervention

Only one of the 61 participants reported an adverse event during MQT-SH. Participants experienced dyspepsia after the second MQT-SH session. The dyspepsia was moderate in severity, unrelated to the intervention, and resolved without any specific treatment.

## 4 Discussion

This study investigated the effectiveness and safety of the MQT-SH in patients diagnosed with both Hwabyung and depressive disorders. Additionally, this study investigated the therapeutic mechanism of the MQT-SH and the effects of home practice on intervention changes.

This study suggests that a standardized program combining mindfulness and qigong can alleviate major symptoms in patients with Hwabyung and depressive disorders. MQT-SH significantly reduced Hwabyung and depressive symptoms. These findings are consistent with previous studies showing that mindfulness and qigong alleviate Hwabyung and depressive symptoms. According to previous studies, mindfulness meditation reduced Hwabyung symptoms in middle-aged women (33) and a qigong-based stress reduction program reduced Hwabyung personality and symptoms in adults who complained of subjective stress (43). A meta-analysis by Hofmann et al. (69) found that mindfulness-based treatment improved depressive symptoms in patients with depressive disorders with a high effect size (Hedges’*g* = 0.95). Another meta-analysis by Liu et al. (58) found that qigong and tai chi reduced depressive symptoms to medium effect sizes (Cohen’s *d* = 0.48). In this study, the effect size of MQT-SH on Hwabyung and depression was high, suggesting significant clinical efficacy of MQT-SH in patients with psychosomatic disorders.

This study showed that MQT-SH alleviated secondary symptoms, such as anxiety, anger, and increased subjective vitality, in patients with Hwabyung and depressive disorders. Hwabyung and depressive disorder are highly comorbid with anxiety disorder (13, 70), and Hwabyung is accompanied by somatic symptoms related to anger (1, 2). Specifically, MQT-SH alleviated anxiety and anger symptoms and reduced anger suppression in patients with Hwabyung and depressive disorders. These findings are consistent with previous studies showing that various mind-body interventions reduce anxiety and anger symptoms and improve dysfunctional anger expression styles (43, 69, 71). Given that Hwabyung is associated with physical and psychological symptoms of anger suppression such as chest tightness and resentment, these findings suggest that the MQT-SH may address the cognitive-behavioral style underlying Hwabyung.

This study explored the therapeutic mechanisms of MQT-SH for emotion regulation. Physical and psychological vitality may be the therapeutic mechanisms of qigong-based programs for Hwabyung and depression. Specifically, the effectiveness of the MQT-SH on Hwabyung symptoms was mainly mediated by physical vitality, whereas the effectiveness of MQT-SH on depression was mediated only by psychological vitality. These findings may be due to differences in the disease characteristics of Hwabyung and depression and suggest that while physical and psychological vitality should be improved to treat psychosomatics such as Hwabyung, psychological vitality should be improved primarily to treat mood disorders such as depression. Previous studies have suggested that mindfulness, cognitive flexibility, and self-compassion are key mechanisms in mind-body interventions for emotion regulation (72). These mechanisms are associated with the features of mindfulness practices that emphasize the being mode. In contrast, qigong is a unique practice that promotes physical and mental health by cultivating and regulating vital energy and has a more active goal beyond the being mode. This study expands our understanding of the different mechanisms of mind-body interventions by investigating qigong-related therapeutic mechanisms.

Research on the therapeutic mechanisms of MQT-SH is still in its early stages. Future studies should conduct three-arm randomized controlled trials, including mindfulness-based programs and measure alternative mechanisms, including mindfulness and self-compassion, to compare the relative effect sizes between mediators. MQT-SH is a program that combines mindfulness and qigong; therefore, it may share mechanisms with mindfulness-based programs. Nevertheless, a comparison with programs that focus on mindfulness may reveal the unique mechanisms of qigong practice.

This study found that the effectiveness of the MQT-SH were largely maintained even after the end of the intervention. However, the follow-up period was relatively short (six weeks). Considering that previous studies performed a long-term follow-up of 6 months or 1 year (73, 74), a long-term follow-up of 6 months or more is necessary in future studies. Long-term follow-up studies can provide evidence for ongoing monitoring by examining how long the effectiveness of an intervention is maintained.

Meanwhile, this study determined that the effectiveness of MQT-SH on depression, anxiety, and subjective vitality were decreased at 6-week follow-up. Depressive disorder is a condition with a high relapse rate (75). Therefore, uncontrolled stressful events following intervention may re-exacerbate depression and related symptoms. Similarly, previous studies have demonstrated that the effectiveness of mindfulness or qigong training on depression, anxiety, and fatigue do not last longer than 3 months (76, 77), suggesting that the effects of mind-body interventions on any outcome may be short-term. However, the findings of this study on the follow-up effects possess the limitation of low internal validity, as they were the results of an analysis of the EG alone, thus making it difficult to draw definitive conclusions. For example, significant historical events may have occurred between intervention and follow-up. Alternatively, the placebo effect may have influenced follow-up self-reports by the EG participants, which may have overestimated the persistence of the intervention effect or underestimated it because the intervention ended. Therefore, future studies should compare active CG to investigate the follow-up effects of MQT-SH.

Previous studies investigating the effects of home practice have yielded mixed findings; therefore, this study conducted a replication verification of the effects of home practice. This study determined that the frequency and duration of home practice did not exhibit a significant correlation with any outcome change. This finding could be interpreted to mean that informal individual practices outside of formal programs provided by professionals have little effect on intervention change. However, this conclusion should be interpreted cautiously, as the methods and procedures used to assess home practices may have influenced these findings (54). For example, in a study by Carmody and Baer (52) in which the frequency and duration of home practice were recorded daily, the amount of home practice was significantly related to symptom improvement. In contrast, other studies that retrospectively recorded the total amount of home practice after the end of the intervention observed no significant relationship between home practice and symptom improvement (53, 54). Retrospective recordings may underestimate or overestimate actual home practice, and the reliability of the recordings may be negatively affected as the gap between actual practice and assessment time increases. In this study, the participants were asked to record each home practice session; however, the recordings were checked weekly. This assessment procedure does not rule out the possibility of retrospective recording. Therefore, future studies should design methods that reliably and validly evaluate the actual amount of home practices. One alternative is to design experience-sampling methods that collect real-time experiences using mobile devices, as this approach can reduce retrospective recording errors.

MQT-SH can be considered a low-risk intervention. In this study, only one of the 61 participants reported an adverse event. The adverse event reported in this study was moderate in severity, nonpersistent, and resolved. A previous study has shown that more than 74% of outpatients in psychiatric hospitals experience antidepressant side effects, and more than two-thirds of them experienced moderate or severe side effects (78). Accordingly, the possibility and severity of adverse events in the MQT-SH were considered significantly lower than those in the medication. Other studies have reported that the percentage of people who experienced adverse events while practicing mind-body training ranged from 1.9% to 25.4% (79–81), suggesting that MQT-SH carries as low of a risk as that of other mind-body trainings.

Although this study suggests that MQT-SH is a relatively safe intervention compared to medication, it does not mean that it is completely risk-free. If the intervention is provided by an instructor who does not possess the appropriate experience or qualifications, it may result in adverse events such as delusions, hallucinations, fatigue, and pain (82, 83). Therefore, training healthcare professionals may be necessary to safely implement mind-body interventions in clinical settings.

### 4.1 Limitations

This study had some limitations. First, although this study was designed as a block randomized controlled trial, baseline homogeneity across the groups was violated in several outcomes. Although ANCOVA was conducted to statistically address group heterogeneity, the severity of symptoms at baseline may have affected intervention adherence, leading to intervention changes. In this study, attendance was significantly higher in the EG than it was in the CG. Considering that the EG exhibited higher Hwabyung and depression scores at baseline than those of the CG, it is anticipated that more severe the symptoms resulted in higher motivation for treatment, and this may lead to higher adherence.

Second, the allocation information of this study was blinded to the participants and researchers. However, while the blinding of the assessors was successful, it is suspected that the blinding of the participants and instructor failed due to the nature of the intervention. As the mind-body intervention was conducted through the interaction between the participants and the instructor, compared to the CG assigned to the wait-list control that did not receive any treatment, the participants in the EG were aware of the active intervention being carried out in their group. Failure of blinding such as the placebo effect can threaten the internal validity of a study. Therefore, future studies should conduct comparisons to active CG.

Third, this was a single-center trial. A single-center trial possesses the advantage of better compliance with the study protocol than does a multicenter trial. However, as a single-center trial was conducted at one institution, it was difficult to recruit a large sample, and this may result in implications for generalizability and external validity. Therefore, future studies should design multicenter trials and recruit larger samples to increase the reliability of the results.

Fourth, this study did not consider demographic variables sufficiently. Demographic variables such as education level, occupation, socioeconomic status, and past experience of mind-body training may affect sampling and intervention adherence. Future studies should include various demographic variables that may affect intervention outcomes.

## 5 Conclusions

This study provides evidence that MQT-SH could be an effective and safe treatment for patients with Hwabyung and depressive disorders. MQT-SH significantly alleviated Hwabyung, depression, anxiety, and anger, and improved subjective vitality in patients with Hwabyung and depressive disorders compared to no treatment. As a therapeutic mechanism, subjective vitality mediated the effectiveness of MQT-SH for Hwabyung and depression. Also, only one of the 61 patients who underwent MQT-SH reported an adverse event. Therefore, MQT-SH could be considered as a novel complementary and alternative treatment for psychosomatic and mood disorders.

## Data Availability

The raw data supporting the conclusions of this article will be made available by the authors, without undue reservation.

## Supplementary material

Supplementary Material 2 Mediation model for the therapeutic mechanism of MQT-SH HS, Hwabyung Scale; HCT, Hwabyung Comprehensive Test; HDRS, Hamilton Depression Rating Scale; IVS = Integrative Vitality Scale.
