# Supplementary figures and images for: Effectiveness of Mindfulness and Qigong Training for Self-Healing in patients with Hwabyung and depressive disorder: a randomized controlled trial
Source: Front Psychiatry. 2025 Jun 13;16:1508937. doi: 10.3389/fpsyt.2025.1508937 (PMC12202437; doi:10.3389/fpsyt.2025.1508937)

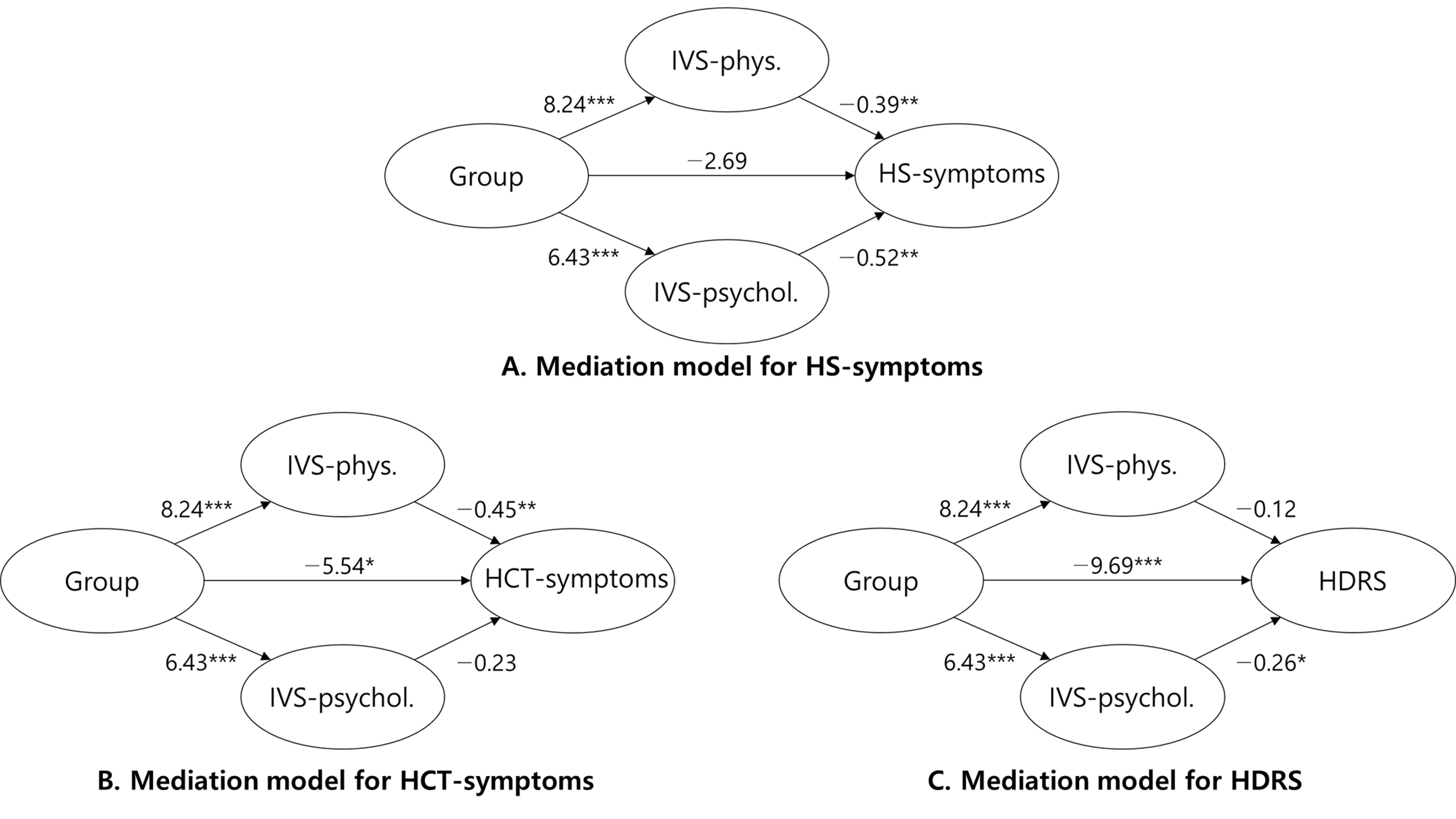

Supplement: Supplementary Material 2 — Mediation model for the therapeutic mechanism of MQT-SH HS, Hwabyung Scale; HCT, Hwabyung Comprehensive Test; HDRS, Hamilton Depression Rating Scale; IVS = Integrative Vitality Scale. [file Image1.tif]
