# Supplementary material for: Effectiveness of Mindfulness and Qigong Training for Self-Healing in patients with Hwabyung and depressive disorder: a randomized controlled trial
Source: Front Psychiatry. 2025 Jun 13;16:1508937. doi: 10.3389/fpsyt.2025.1508937 (PMC12202437; doi:10.3389/fpsyt.2025.1508937)
Supplement: Supplementary file 2 [file Table1.docx]

Supplementary Material 1. Results of mediation analysis

| **DV** | **Route** | | **B** | **SE** | **t / 95% CI** |
| --- | --- | --- | --- | --- | --- |
| HS-symptoms | Total | Group ⇒ HS-symptoms | −9.23 | 2.06 | −3.94^**^ |
|  | Direct | Group ⇒ HS-symptoms | −2.69 | 2.06 | −1.31 |
|  | Indirect | Group ⇒ IVS-phys. ⇒ HS-symptoms | −3.17 | 1.46 | [−6.70, −0.82] |
|  |  | Group ⇒ IVS-psychol. ⇒ HS-symptoms | −3.37 | 1.59 | [−6.99, −0.76] |
| HCT-symptoms | Total | Group ⇒ HCT-symptoms | −10.76 | 2.29 | −4.71^***^ |
|  | Direct | Group ⇒ HCT-symptoms | −5.54 | 2.23 | −2.48^*^ |
|  | Indirect | Group ⇒ IVS-phys. ⇒ HCT-symptoms | −3.74 | 1.87 | [−8.18, −0.66] |
|  |  | Group ⇒ IVS-psychol. ⇒ HCT-symptoms | −1.48 | 1.44 | [−5.31, 0.63] |
| HDRS | Total | Group ⇒ HDRS | −12.31 | 1.40 | −8.81^***^ |
|  | Direct | Group ⇒ HDRS | −9.69 | 1.46 | −6.66^***^ |
|  | Indirect | Group ⇒ IVS-phys. ⇒ HDRS | −0.96 | 0.77 | [−2.77, 0.32] |
|  |  | Group ⇒ IVS-psychol. ⇒ HDRS | −1.66 | 0.96 | [−4.09, −0.14] |
| HS, Hwabyung Scale; HCT, Hwabyung Comprehensive Test; HDRS, Hamilton Depression Rating Scale; IVS, Integrative Vitality Scale  Number of bootstrap samples for bias corrected bootstrap confidence intervals = 10,000  ^***^p<0.001, ^**^p<0.01, ^*^p<0.05 | | | | | |
